# The “Dental Digest” and the “Faculties”

**Published:** 1901-08

**Authors:** 


### Editorial.

### THE "DENTAL DIGEST" AND THE "FACULTIES."

The July number of the *Dental Digest* contains an editorial on "The Duty of the Faculties Association." This is by no means a novel subject, for the readers of this journal can safely and surely look forward to an editorial at this season of the year scarifying the Association of Dental Faculties and dental colleges in general. The editor of the *Digest* would, seemingly, approach the fall months in an unhappy frame of mind if this duty had been neglected.

While this earnest persistence in a course supposed to be right is to be commended, there are some connected with college work who fail to understand the motive force that continually drives the editor of the *Digest* into desperation during the high-temperature days. It is understood that the editor is never quite happy unless he has something to say against dental colleges. Having, however, apparently exhausted his mental efforts in this direction, he repeats his editorial of June last year in this July number, adding a few words by way of refreshment to his readers.

The motive which brought forth this editorial was found in the address of the President before the Illinois State Dental Society and the discussion which followed. The main topic of this address was, of course, dental education. Whenever everything else fails this is an ever inexhaustible topic. The remarks upon this paper, if reported correctly, do not reflect much credit upon the participants. One arrives at this conclusion: "If the Association [Dental Faculties Association] sees fit to continue whitewashing incompetent colleges, as it has done in the past, and to receive as members schools which are verging on the disreputable, then the sooner it disbands the better it will be for dental education." Again, he says, "I am not willing to remain quiet longer and be classed as a member of the Faculties Association when it admits and retains some of the schools that are in it to-day."

The distinct charge here made, if true, should be met by the Association, and it is to be hoped that before this reaches our readers this will have been done. That there are weak schools connected with the Faculties may be true. If so, the fault lies not with the Association, but with the boards of examiners of the several States primarily, and with the committees, of which there are two, one preliminary and one final, appointed to examine each school before it can be considered. It is assumed, without any fear of successful contradiction, that no body of men have more carefully lived up to their rules in this respect. While this is true it is equally certain that in this, as in all associations, all churches, all governments, are to be found persons ready to thwart the best efforts of those in control. The college without a degenerate would be an anomaly. A profession without an empirical impostor would be an organization that has never yet existed. The gentleman who made the remarks quoted, while nominally a member of the Faculties, through his college, has never taken any active part in its proceedings, and hence is quite incompetent to form an opinion.

The writer, early in the history of this body, was made chairman of a standing committee to receive complaints made against colleges violating rules. It was originally named the Ad Interim Committee, as it was supposed to be actively at work during the period between the sessions. For years the writer served without fear or favor in this capacity, and during that time there was never any "whitewashing" done, not even of the highest dental colleges in the land, as some found to their cost. While the attempts made to violate rules were frequent, they were invariably met and the parties disciplined, but of this the outside dental world knew nothing, and hence inferred that the Faculties accepted members without proper examination, and then permitted them to go on in their own way regardless of rules or professional requirements.

The Association of Faculties since its organization in 1884 has had a serious task given it to perform. Those who were familiar with the dental profession prior to that time can appreciate the results of this labor. The handling of the many questions that have from time to time come before this body required the best judgment and most tactful consideration. The Association has done a great work, and is still doing it, in spite of clamor outside from parties ignorant or woefully blind to the progress made in dental education through its efforts.

The writer is not attempting here an apology for the Association of Faculties. It needs none. With all its imperfections as an organization it has combined the dental educational forces, to the end that a higher standard has been attained in this country than could possibly have been anticipated seventeen years ago, and which could not have been accomplished without its aid.

The editor of the *Digest* settles this whole question for himself when he says, "Not only is the National Association of Dental Faculties rapidly losing caste in this country and abroad, but American dental diplomas are becoming a laughing-stock in Europe." This coming from Chicago and the State of Illinois, that has through its lax laws made American diplomas a byword in Europe, is the most remarkable manifestation of what the slang world denominates gall that it has been the writer's privilege to see in print for some time. No one knows better than the editor of the *Digest* that it was through the efforts of the Foreign Relations Committee of the National Association of Dental Faculties, and the money from its treasury, that these foreign (not American) diploma mills were crushed out and some of the proprietors landed in jail. There is a vast work to be done in starting the so-called school men of Illinois upon the right path, and we would suggest that the editor of the *Digest* turn his great mental activities in that direction, and leave his midsummer stereotyped editorial for a future day and generation that may need it.

To those outside of the Association of Faculties who claim everything in the way of educational progress it may be said that there is a modest path that worthy men prefer to follow, and the writer would recommend them to seek it. In the mean time the

Association of Faculties will go on its way, will not disband, but quietly and surely build up the dental educational structure until its proportions will be such that even the most rabid opponent will be silenced and the ingrained jealousy of the *Digest* will have died a natural death.

